# Myeloid cell-derived catecholamines influence bone turnover and regeneration in mice

**DOI:** 10.3389/fendo.2022.997745

**Published:** 2022-09-15

**Authors:** Melanie R. Kuhn, Melanie Haffner-Luntzer, Elena Kempter, Stefan O. Reber, Hiroshi Ichinose, Jean Vacher, Anita Ignatius, Miriam E. A. Tschaffon-Müller

**Affiliations:** ^1^ Institute of Orthopedic Research and Biomechanics, University Medical Center Ulm, Ulm, Germany; ^2^ Laboratory for Molecular Psychosomatics, Department of Psychosomatic Medicine and Psychotherapy, University Ulm, Ulm, Germany; ^3^ School of Life Science and Technology, Tokyo Institute of Technology, Yokohama, Japan; ^4^ Department of Medicine, Institut de Recherches Cliniques de Montréal, Montréal, QC, Canada

**Keywords:** tyrosine hydroxylase, fracture healing, inflammation, bone growth, bone metabolism

## Abstract

Catecholamine signaling is known to influence bone tissue as reuptake of norepinephrine released from sympathetic nerves into bone cells declines with age leading to osteoporosis. Further, β-adrenoceptor-blockers like propranolol provoke osteoprotective effects in osteoporotic patients. However, besides systemic adrenal and sympathetic catecholamine production, it is also known that myeloid cells can synthesize catecholamines, especially under inflammatory conditions. To investigate the effects of catecholamines produced by CD11b^+^ myeloid cells on bone turnover and regeneration, a mouse line with specific knockout of tyrosine hydroxylase, the rate-limiting enzyme of catecholamine synthesis, in CD11b^+^ myeloid cells (TH^flox/flox^/CD11b-Cre^+^, referred to as TH^CD11b-Cre^) was generated. For bone phenotyping, male mice were sacrificed at eight and twelve weeks of age and harvested bones were subjected to bone length measurement, micro-computed tomography, fluorescence-activated cell sorting of the bone marrow, gene expression analysis, histology and immunohistochemistry. Support for an age-dependent influence of myeloid cell-derived catecholamines on bone homeostasis is provided by the fact that twelve-week-old, but not eight-week-old TH^CD11b-Cre^ mice, developed an osteopenic phenotype and showed increased numbers of neutrophils and T lymphocytes in the bone marrow, while CCL2, IL-6, IL-4 and IL-10 mRNA expression was reduced in sorted myeloid bone marrow cells. To investigate the influence of myeloid cell-derived catecholamines on fracture healing, mice received a diaphyseal femur osteotomy. Three days post-fracture, immunohistochemistry revealed an increased number of macrophages, neutrophils and cytotoxic T lymphocytes in the fracture hematoma of TH^CD11b-Cre^ mice. Micro-computed tomography on day 21 showed a decreased tissue mineral density, a reduced bone volume and less trabeculae in the fracture callus indicating delayed fracture healing, probably due to the increased presence of inflammatory cells in TH^CD11b-Cre^ mice. This indicates a crucial role of myeloid cell-derived catecholamines in immune cell-bone cell crosstalk and during fracture healing.

## Introduction

Bones are a crucial part of the musculoskeletal system, allowing locomotion and providing mechanical protection of the internal organs. Furthermore, bone tissue is involved in metabolic functions such as calcium storage and hematopoiesis (1, 2). Different cell types are essential for development, remodeling and regeneration of bone. The most important cell types are bone forming osteoblasts, bone resorbing osteoclasts, osteocytes embedded in the bone matrix and cartilage forming chondrocytes (1, 3). Bone is continuously remodeled to repair microdamage and to respond to changing mechanical stimuli during the development of an organism. Especially trabecular bone is highly remodeled. The process of bone remodeling needs to be well balanced: excessive bone resorption by osteoclasts without sufficient neoformation of bone may lead to osteoporosis, whereas excessive bone formation versus resorption results in increased bone mass (1). The physiological balance in bone formation and resorption is controlled by different factors including biomechanical load, hormones, cytokines and chemokines (1). In recent years, it became more and more evident that also neurotransmitters like catecholamines strongly influence bone metabolism. Striking clinical evidence is given by the fact that catecholamine-producing tumors increase the risk for osteoporosis and osteoporotic fractures (4) and that β-adrenoceptor-blockers like propranolol provoke osteoprotective effects in osteoporotic patients (5). Furthermore, it was shown in preclinical studies that the bone tissue expression of the norepinephrine transporter (NET), which is involved in the reuptake of released norepinephrine (NE), declines with increasing age, leading to a local surplus of catecholamines (6, 7). This indicates an important role of local bone NE signaling in the manifestation of age-related osteoporosis. On a cellular level, it is known that different subtypes of adrenoceptors (ARs), namely α_1_, α_2_, β_1_, β_2_ and β_3_, are expressed by osteoblasts, osteocytes, osteoclasts and chondrocytes, depending on the species investigated (8–10). Importantly, these different ARs appear to mediate distinct effects. In osteoblastic cells, for instance, α_1_-AR signaling increases proliferation and differentiation, while β_2_-AR signaling has opposite effects (9, 11, 12). In detail, while activation of β_2_-AR signaling in osteoblasts increased receptor activator of NF-κB ligand (RANKL) expression, thereby stimulating osteoclastogenesis, blocking of β-AR signaling increased osteoblast differentiation and decreased RANKL expression (9), explaining the osteoprotective effects of beta-blockers in patients (5, 13). Further evidence for a negative influence of β_2_-AR signaling on osteoblasts is given by the fact that β_2_-AR knockout mice displayed increased bone mass (14). β_2_-AR signaling also regulates chondrogenic differentiation (15, 16). The expression of early chondrogenic markers, including SRY-box transcription factor 9 (Sox9) and collagen type II, was suppressed by epinephrine or NE treatment, as well as the expression of hypertrophy markers like collagen type X (17, 18). Furthermore, it was shown that conditional deletion of the β_2_-AR in mesenchymal stem cells inhibited progression of osteoarthritis (19) and that NE signaling *via* β-ARs is involved in chondrocyte metabolism under inflammatory conditions (20, 21). Dopamine signaling was also shown to influence bone cells, as mice treated with the dopamine antagonist Risperidone display increased osteoclastogenesis (22) and dopaminergic stimulation of bone marrow-derived mesenchymal stem cells attenuated osteogenic differentiation (23).

In conclusion, there is strong evidence that catecholamine signaling influences bone metabolism. Besides systemic adrenal and sympathetic catecholamine production, it is also known that myeloid cells can synthesize catecholamines, especially under inflammatory and chronic psychosocial stress conditions (24–26). This might directly influence the cellular functioning of the myeloid cells themselves and, indirectly, also other immune cells as well as bone cells, as all these various cell types are located in close proximity in the bone marrow (27). To investigate the effects of catecholamines produced locally by CD11b^+^ myeloid cells on bone homeostasis and regeneration, a mouse line with specific knockout of tyrosine hydroxylase (TH), the rate-limiting enzyme of catecholamine synthesis (28), in CD11b^+^ myeloid cells (TH^flox/flox^/CD11b-Cre^+^, referred to as TH^CD11b-Cre^) was generated. We analyzed the bone phenotype of eight- and twelve-week-old adolescent male mice to investigate the role of myeloid cell-derived catecholamines on bone development and juvenile bone metabolism as well as on adult bone metabolism in more mature mice and regeneration, respectively. In summary, we demonstrated an age-dependent influence of myeloid cell-derived catecholamines on bone metabolism and a crucial role during fracture healing.

## Methods

### Animals

Male TH^flox/flox^/CD11b-Cre^+^ mice (C57BL/6 background, referred to as TH^CD11b-Cre^) with specific knockout of tyrosine hydroxylase in CD11b^+^ myeloid cells (TH-KO) were obtained by crossing male CD11b-Cre^+^ mice (provided by Dr. Jean Vacher, Institut de Recherches Cliniques de Montréal, Québec, Canada) with female TH^flox/flox^ mice (provided by Prof. Dr. Hiroshi Ichinose, Tokyo Institute of Technology, Japan). As control, male TH^flox/flox^/CD11b-Cre^-^ mice (C57BL/6 background, referred to as TH^flox/flox^), generated by backcrossing female CD11b-Cre mice with male TH^flox/flox^ mice, were used. Since the CD11b-Cre transgene is inserted into the Y-chromosome in our mouse line, only male mice could be used for this study. Genotyping *via* polymerase chain reaction (PCR) was performed with digested ear punches using the following primers: *CD11b-Cre* (Forward primer sequence (F): 5’-AATGCTTCTGTCCGTTTGC-3’, Reverse primer sequence (R): 5’-CGGCAACACCATTTTTTCTG-3’), *TH flox* (F: 5’-ATTTGCCCAGTTCTCCCAG-3’, R: 5’-AGAGATGCAAGTCCAATGTC-3’) and *TH KO* (F: 5’-GGCGTATCGCCAGCGCCGG-3’, R: 5’-CCCCAGAGATGCAAGTCCAATGTC-3’). Specificity of the knockout was verified by analyzing TH expression in CD11b^+^ and CD11b^-^ cells by FACS (**Supplementary Figure 1**). Up to five mice were housed together in one cage and lived under constant standard mouse conditions in the laboratory including 12 h light/dark per day, 60% humidity and 22°C room temperature. Mice were provided with standard mouse feed (ssniff, V 1534 - 000 R/M-H) and tap water ad libitum. The animal study was carried out in accordance with the ARRIVE guidelines 2.0 (29) and was approved by the Federal Animal Care and Use Committee (Regierungspräsidium Tübingen, Germany).

### Experimental design

To investigate the effects of the CD11b^+^-cell specific knockout of TH on bone and bone marrow immune phenotype under physiological conditions, male TH^CD11b-Cre^ and TH^flox/flox^ mice were sacrificed at either 8 or 12 weeks of age (n = 8 per group and time point). After respiratory arrest induced with an overdose of isoflurane (Baxter), intact femora and tibiae were removed. The bone lengths of the left femora and tibiae were measured with a manual caliper (Horex). To investigate the influence of the TH-KO on fracture healing, 10-11-week-old TH^CD11b-Cre^ and TH^flox/flox^ mice (n = 6 per group) received a standardized unilateral diaphyseal osteotomy of the right femur stabilized by an external fixator (RISystem) as described previously (30). 10-11 weeks of age were chosen because at this age, the mice reached the minimal femur length that the external fixator can be safely mounted. 3 and 21 days after osteotomy, mice were euthanized by brief CO_2_ inhalation followed by decapitation and fractured femora were harvested. The experimental design was approved by the local ethical committee (No. 1437, No. O.135-7, Regierungspräsidium Tübingen, Germany). We have chosen 3 and 21 days as endpoints for our analysis, because at 3 days after fracture many important immune cells are highly present in the fracture callus (especially neutrophils and T lymphocytes), while 21 days after fracture is the best time point for analyzing bone formation after fracture as an important outcome measure for the success of fracture healing.

### Enzyme immunoassay

Directly after euthanasia, blood samples were collected from unfractured 12-week-old mice by puncture of the heart. The blood samples were incubated for at least 30 min on ice and serum was harvested after centrifugation for 7 min at 13000 g. Rat/Mouse PINP Enzyme immunoassay (EIA) (IDS) and RatLaps (CTX-I) EIA (IDS) were performed according to the manufacturer’s instructions and measured at 450 nm and a reference of 650 nm using the UV spectrometer Spark (Tecan). Analysis was performed using a 4-parametric logistic standard curve.

### Fluorescence-activated cell sorting

Bone marrow immune cells of the left intact tibiae were quantified using fluorescence-activated cell sorting (FACS). After bone marrow isolation and erythrolysis, samples were centrifuged (5804 R, Eppendorf) twice for 5 min at 1800 rpm and room temperature with 10 mL phosphate-buffered saline (Thermo Fisher Scientific). 100 μL of each sample were added to a 96-well plate (Greiner Bio-One) and incubated for 30 min on ice in the dark with the following fluorescent labeled antibodies: rat anti-mouse CD11b Alexa Fluor 700 (1:400, 56-0112-80, eBioscience), rat anti-mouse Ly6G V450 (1:400, 560603, BD Biosciences), rat anti-mouse F4/80 FITC (1:50, 11-4801-82, eBioscience), Armenian hamster anti-mouse CD3e PE-Cyanine7 (1:100, 25-0031-82, eBioscience), rat anti-mouse CD8a APC (1:800, 17-0081-81, eBioscience) and rat anti-mouse CD4 APC-eFluor 780 (1:200, 47-0041-82, eBioscience). Rat IgG2b κ Alexa Fluor 700 (1:400, 56-4031-80, eBioscience), rat IgG2a κ V450 (1:400, 560377, BD Bioscience), rat IgG2a κ FITC (1:50, 11-4321-82, eBioscience), Armenian hamster IgG PE-Cyanine7 (1:100, 25-4888-82, eBioscience), rat IgG2a κ APC (1:800, 17-4321-81, eBioscience) and rat IgG2b κ APC-eFluor 780 (1:200, 47-4031-82, eBioscience) were used as isotype controls. After washing with FACS buffer, samples were fixed for 10 min in 0,5% formalin in phosphate-buffered saline (Thermo Fisher Scientific). For the subsequent intracellular staining of tyrosine hydroxylase (TH), cell membranes were permeabilized with 0,1% Triton X-100 (Sigma-Aldrich) in FACS buffer. Rabbit anti-mouse TH (1:200, AB152, Merck Millipore) as primary antibody and goat anti-rabbit PE (1:500, ab72465, abcam) as secondary antibody and negative control were added to the samples for 20 min. CD11b^+^ myeloid immune cells, Ly6G^+^ neutrophils, F4/80^+^ macrophages, CD3e^+^ T lymphocytes, CD3e^+^CD8a^+^ cytotoxic T lymphocytes and CD3e^+^CD4^+^ T helper lymphocytes were quantified with BD FACSLyric Flow Cytometer (BD Bioscience) and FlowJo software (v10).

### μCT analysis

Left femora of unfractured mice and right fractured femora of mice euthanized 21 days after fracture were subjected to micro-computed tomography (μCT) using a SkyScan 1172 (Bruker) scanning device. µCT scan was performed with 50 kV, 200 mA and a voxel resolution of 8 μm. The trabecular parameters of the intact femora (trabecular tissue mineral density (Tb.TMD), bone volume to tissue volume ratio (BV/TV), trabecular number (Tb.N), trabecular thickness (Tb.Th) and trabecular separation (Tb.Sp)) were measured in the femoral metaphysis in volume of interest 1 (VOI 1) located 360 µm proximal to the distal femoral growth plate with a height of 280 µm. The cortical parameters of the intact femora (cortical tissue mineral density (C.TMD) and cortical thickness (C.Th)) were quantified in the femoral mid-diaphysis in volume of interest 2 (VOI 2) placed under the third trochanter with a height of 80 µm. The parameters of the fractured femora (TMD, BV/TV, Tb.N, Tb.Sp) were analyzed in the periosteal and endosteal callus between the two inner pinholes of the external fixator. Two phantoms containing a defined amount of hydroxyapatite (250 mgHA/cm^3^ and 750 mgHA/cm^3^) were scanned together with the femora to quantify the tissue mineral density (TMD). For Tb.TMD, the threshold to differentiate between mineralized and non-mineralized tissue was set at 394 mgHA/cm^3^. For C.TMD and the TMD of the fracture callus, the threshold was set at 642 mgHA/cm^3^. μCT analysis was carried out in accordance with the guidelines of the American Society for Bone and Mineral Research (ASBMR) (31).

### Histology

After fixation for 48 h in 4% formaldehyde (Otto Fischar), right intact femora were embedded in paraffin (McCormick-Paraplast Plus) for decalcified histology. Left intact femora underwent undecalcified histology *via* embedding in methylmethacrylate (Carl Roth). 4-5 μm thin longitudinal sections of the decalcified right intact femora were stained with Safranin O/Fast Green to quantify the osteoblast number per bone perimeter (N.Ob/B.Pm) and the osteoblast surface per bone surface (Ob.S/BS) in the distal femoral metaphysis (region of interest (ROI): rectangular area (length: 480 µm, width: distance between the cortices) located 960 µm proximal to the distal femoral growth plate). Tartrate-resistant acid phosphatase-(TRAP)-staining was used for the quantification of the osteoclast number per bone perimeter (N.Oc/B.Pm) and the osteoclast surface per bone surface (Oc.S/BS) in the same ROI. 7 μm thin longitudinal sections of the undecalcified left intact femora were subjected to Von-Kossa-staining for the determination of the distal femoral growth plate thickness. All parameters were evaluated using the microscope Axiophot (Carl Zeiss) and the software OsteoMeasure 7 (v4.2.1.0, Osteo-Metrics). Osteoblasts and osteoclasts were quantified at a magnification of 200, the distal femoral growth plate thickness was determined at 50-fold magnification.

### Immunohistochemistry

Right intact and fractured femora were fixed for 48 h in 4% formaldehyde (Otto Fischar) and underwent decalcified histology *via* embedding in paraffin (McCormick-Paraplast Plus). 4-5 μm thin longitudinal sections were used for the immunohistochemical staining of Ly6G^+^ neutrophils (rat anti-mouse Ly6G, 1:300, 127632, BioLegend), F4/80^+^ macrophages (rat anti-mouse F4/80, 1:500, MCA497GA, Bio-Rad Laboratories) and CD8^+^ cytotoxic T lymphocytes (rabbit anti-mouse CD8, 1:500, bs-0648R, Bioss). Goat anti-rat IgG-biotin (1:200, A10517, Invitrogen) and goat anti-rabbit IgG-biotin (1:200, B2770, Invitrogen) were used as secondary antibodies and species-specific non-targeting immunoglobulins (rat IgG, 1:200 for F4/80, 1:120 for Ly6G, 401901, BioLegend and rabbit IgG, 1:500, 011-000-003, Jackson ImmunoResearch) as negative controls. First, the paraffin-embedded sections were deparaffinized, rehydrated and demasked by incubation in citrate buffer. After blocking with 3% peroxidase (Merck), samples were incubated with 5% (F4/80) or 10% (Ly6G, CD8) goat serum (Jackson ImmunoResearch) in washing buffer at room temperature (F4/80) or at 37°C (Ly6G, CD8). One hour later, the primary antibodies and negative controls were placed overnight at 4°C (F4/80, Ly6G) or for 2 h at room temperature (CD8) on the samples followed by incubation with the secondary antibodies for 30 min (CD8) or 1 h (F4/80, Ly6G) at room temperature. The stainings were performed with VECTASTAIN Elite ABC-HRP Kit and NovaRED (Vector Laboratories) as chromogen. For counterstaining, the sections were subjected to hematoxylin (Waldeck). The quantification of the stained immune cells was performed with the microscope Axiophot (Carl Zeiss) and the software OsteoMeasure 7 (v4.2.1.0, Osteo-Metrics). The stained immune cells were counted in a rectangular area (480 µm x 350 µm) in the bone marrow of the intact femora at a magnification of 200. In the fractured femora, neutrophils and cytotoxic T lymphocytes were quantified at 400-fold magnification in, where possible, four rectangular areas, each with a length of 240 µm and a width of 175 µm, located periosteal next to the fracture gap, excluding the cortex. Macrophages were counted, where possible, in four rectangular areas with the same size endosteal next to the fracture gap at the same magnification.

### Gene expression analysis

The PureLink RNA Mini Kit (Thermo Fisher Scientific) was used to isolate RNA from whole intact right tibiae. To obtain RNA from CD11b^+^ and CD11b^-^ bone marrow cells from right and left intact femora and tibiae with the AllPrep DNA/RNA Kit (Qiagen), harvested bone marrow cells were separated by Magnetic Cell Separation (MACS) using CD11b MicroBeads UltraPure (Miltenyi Biotec) and the QuadroMACS Separator (Miltenyi Biotec). After RNA isolation, one-step semi-quantitative Real-Time-Polymerase-Chain-Reactions (RT-PCRs) were performed with the SensiFAST SYBR Hi-ROX One-Step Kit (Bioline) and the QuantStudio 3 RT-PCR System (Thermo Fisher Scientific) according to the manufacturer’s guidelines. The relative gene expression of *CXCL1* (F: 5’-TCTCCGTTACTTGGGGACAC-3’, R: 5’-CCACACTCAAGAATGGTCGC-3’), *CCL2* (F: 5’-GGCTCAGCCAGATGCAGTT-3’, R: 5’-TCTCCAGCCTACTCATTGGGA-3’), *IL6* (F: 5’-TCCTTCCTACCCCAATTTCC-3’, R: 5’-GCCACTCCTTCTGTGACTCC-3’), *IL4* (F: 5’-CCACGGATGCGACAAAAATCA-3’, R: 5’-GTGCATGGCGTCCCTTCTC-3’), *IL10* (F: 5’-GGCAGAGAAGCATGGCCCAGAAATC-3’, R: 5’-ACTCTTCACCTGCTCCACTGCCT-3’), *TNF* (F: 5’-GGCCACCACGCTCTTCTGTCTACT-3’, R: 5’-TGATCTGAGTGTGAGGGTCTGGGC-3’) and *IL1B* (F: 5’-ACAAGGAGAACCAAGCAACG-3’, R: 5’-GGGTGTGCCGTCTTTCATTA-3’) was determined with the delta-delta CT (ΔΔCT) method. *B2M* (F: 5’-ATACGCCTGCAGAGTTAAGCA-3’, R: 5’-TCACATGTCTCGATCCCAGT-3’) was used as the housekeeping gene.

### Statistical analysis

We performed a power analysis for the main outcome measures BV/TV in the intact femur and BV/TV in the fracture callus at day 21 using GPower. Effect size according to previous studies was assumed at 1.40 for intact femurs and 1.35 for fractured femurs. We used the calculation for alpha = 0.05 and power = 0.8, unpaired t-test between two groups. This analysis resulted in a group size of n = 8 and an actual power of 0.8452 and 0.8215. Student’s t-test or Welch t-test were performed for the statistical analysis of the normally distributed data. Mann-Whitney-U test was performed for the analysis of not normally distributed data. Significant differences were assumed at P < 0.05 (* 0.05 > P ≥ 0.01, ** 0.01 > P ≥ 0.001, *** 0.001 > P ≥ 0.0001, **** P < 0.0001). GraphPad Prism (v 8.4.3, GraphPad Software) was used as statistical software. Results are shown as mean and standard deviation (SD). Individual values are presented as black dots. Animal number per group was 6-8. Gene expression analysis was performed with n = 3-4. For non-fractured mice, all mice (n = 8 per group) have been included into the analysis of the main outcome measures (µCT and histology). Some bones had to be excluded for FACS and RNA analysis due to technical problems. For fractured mice, two mice in the CD11b^+^ group had to be excluded from the study due to surgical problems resulting in a too large gap size. Therefore, this group consists only n = 6 mice.

## Results

**Figure 1:**
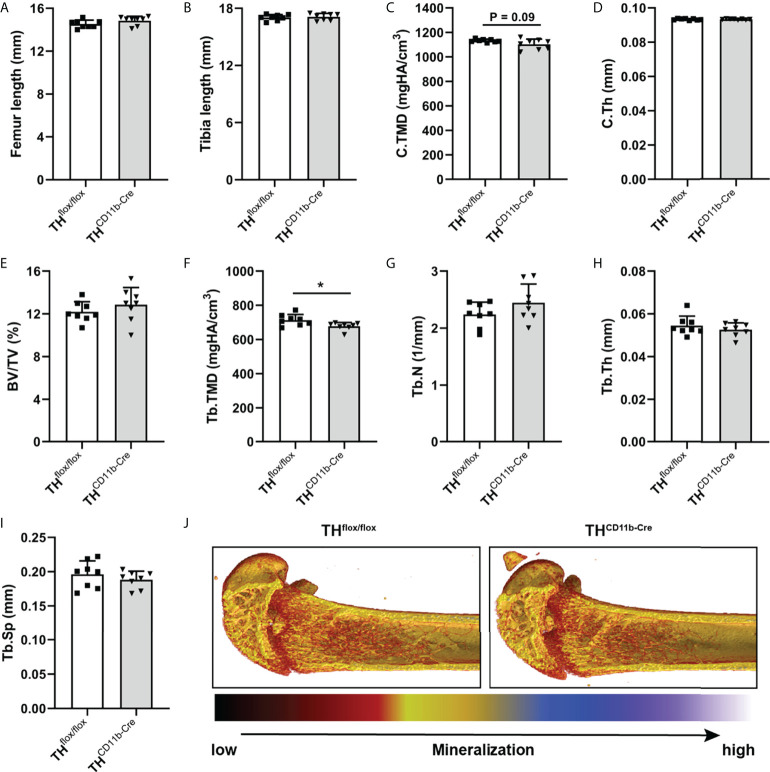
Bone phenotype of eight-week-old TH^CD11b-Cre^ mice. **(A)** Femur and **(B)** tibia length of eight-week-old TH^flox/flox^ and TH^CD11b-Cre^ mice. **(C)** Cortical tissue mineral density (C.TMD), **(D)** cortical thickness (C.Th), **(E)** bone volume to tissue volume ratio (BV/TV), **(F)** trabecular tissue mineral density (Tb.TMD), **(G)** trabecular number (Tb.N), **(H)** trabecular thickness (Tb.Th), **(I)** trabecular separation (Tb.Sp) and **(J)** representative µCT images of femora of eight-week-old TH^flox/flox^ and TH^CD11b-Cre^ mice. n = 8; *P < 0.05.

**Figure 2:**
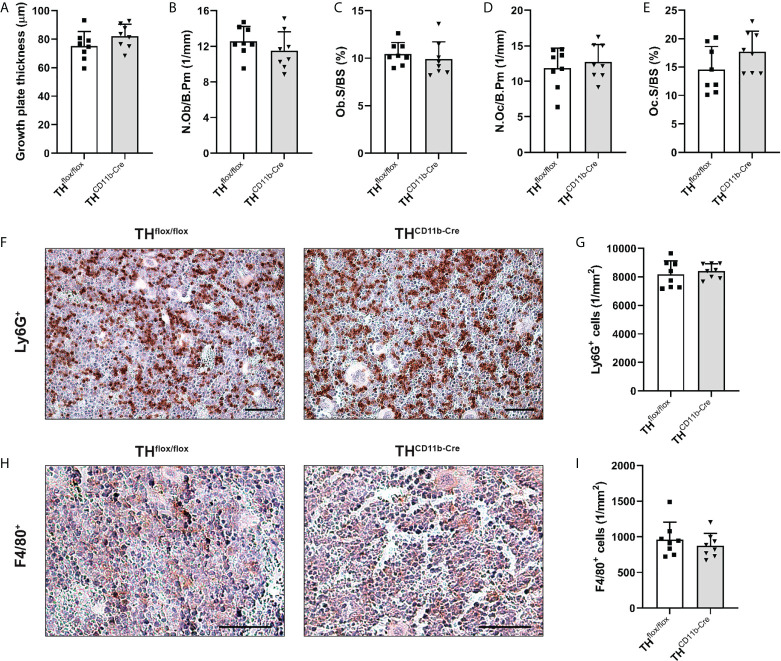
Histological and immunohistochemical analysis of femora of eight-week-old TH^CD11b-Cre^ mice. **(A)** Growth plate thickness, **(B)** number of osteoblasts per bone perimeter (N.Ob/B.Pm), **(C)** osteoblast surface per bone surface (Ob.S/BS), **(D)** number of osteoclasts per bone perimeter (N.Oc/B.Pm), **(E)** osteoclast surface per bone surface (Oc.S/BS), immunohistochemical staining and quantification of **(F, G)** Ly6G^+^ neutrophils and **(H, I)** F4/80^+^ macrophages in femora of eight-week-old TH^flox/flox^ and TH^CD11b-Cre^ mice. Scale bars represent 50 µm. n = 8.

**Figure 3:**
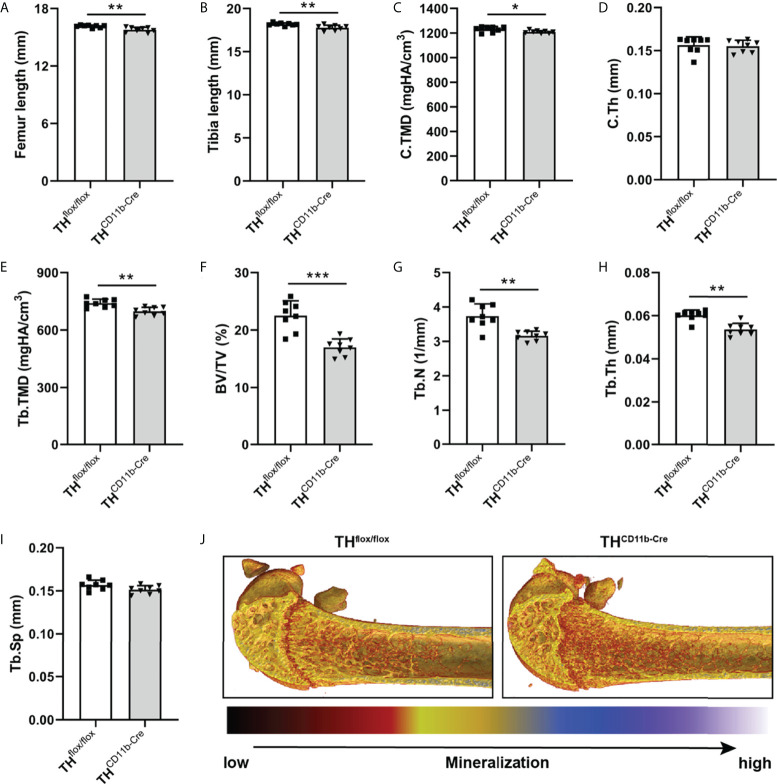
Bone phenotype of twelve-week-old TH^CD11b-Cre^ mice. **(A)** Femur and **(B)** tibia length of twelve-week-old TH^flox/flox^ and TH^CD11b-Cre^ mice. **(C)** Cortical tissue mineral density (C.TMD), **(D)** cortical thickness (C.Th), **(E)** trabecular tissue mineral density (Tb.TMD), **(F)** bone volume to tissue volume ratio (BV/TV), **(G)** trabecular number (Tb.N), **(H)** trabecular thickness (Tb.Th), **(I)** trabecular separation (Tb.Sp) and **(J)** representative µCT images of femora of twelve-week-old TH^flox/flox^ and TH^CD11b-Cre^ mice. n = 8; *P < 0.05; **P < 0.01; ***P < 0.001.

To investigate the effects of the TH-KO in CD11b^+^ myeloid cells on bone metabolism, we analyzed the bone phenotype of TH^CD11b-Cre^ mice, which showed a significant reduction in TH^+^ cells among the CD11b^+^ but not the CD11b^-^ bone marrow cell subpopulation by FACS analysis, compared to TH^flox/flox^ control mice (**Supplementary Figures 1 C, D**). We first analyzed the bone phenotype of eight-week-old mice and found no differences in femur and tibia length (**Figures 1A, B**). Furthermore, there was no difference regarding cortical tissue mineral density (TMD) and cortical thickness (**Figures 1C, D**). Trabecular TMD was significantly reduced in TH^CD11b-Cre^ mice compared to TH^flox/flox^ control mice, while all other trabecular parameters did not differ (**Figures 1E–J**). Trabecular separation did not significantly differ between both groups (**Figures 3I**). Moreover, we also did not detect any differences between the two mouse lines regarding growth plate thickness as well as the number and surface of osteoblasts and osteoclasts in the metaphyseal part of the femur (**Figures 2A–E**). Finally, immunohistochemical staining for the neutrophil marker Ly6G and the macrophage marker F4/80 revealed no genotype-related differences in these immune cell populations in the bone marrow at the age of eight weeks (**Figures 2F–I**).

**Figure 4:**
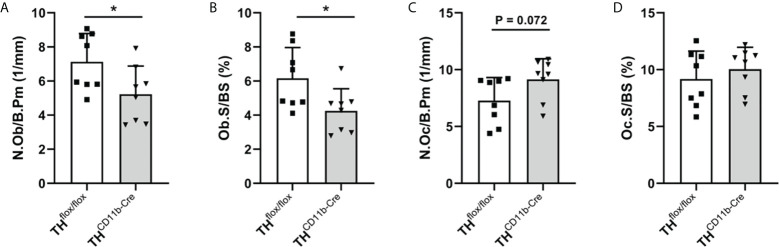
Bone cells in twelve-week-old TH^CD11b-Cre^ mice. **(A)** Number of osteoblasts per bone perimeter (N.Ob/B.Pm), **(B)** osteoblast surface per bone surface (Ob.S/BS), **(C)** number of osteoclasts per bone perimeter (N.Oc/B.Pm) and **(D)** osteoclast surface per bone surface (Oc.S/BS) in femora of twelve-week-old TH^flox/flox^ and TH^CD11b-Cre^ mice. n = 8; *P < 0.05.

**Figure 5:**
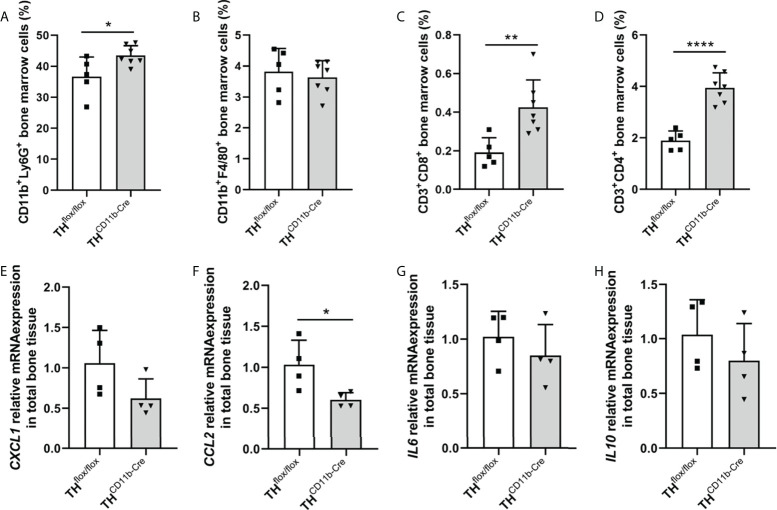
Immune phenotype of twelve-week-old TH^CD11b-Cre^ mice. **(A)** CD11b^+^Ly6G^+^ neutrophils, **(B)** CD11b^+^F4/80^+^ macrophages, **(C)** CD3^+^CD8^+^ cytotoxic T lymphocytes and **(D)** CD3^+^CD4^+^ T helper lymphocytes in the bone marrow of twelve-week-old TH^flox/flox^ and TH^CD11b-Cre^ mice. **(E)** *CXCL1*, **(F)** *CCL2*, **(G)** *IL6* and **(H)** *IL10* relative mRNA expression in whole tibiae of twelve-week-old TH^flox/flox^ and TH^CD11b-Cre^ mice. n = 4-7; *P < 0.05; **P < 0.01; ****P < 0.0001.

**Figure 6:**
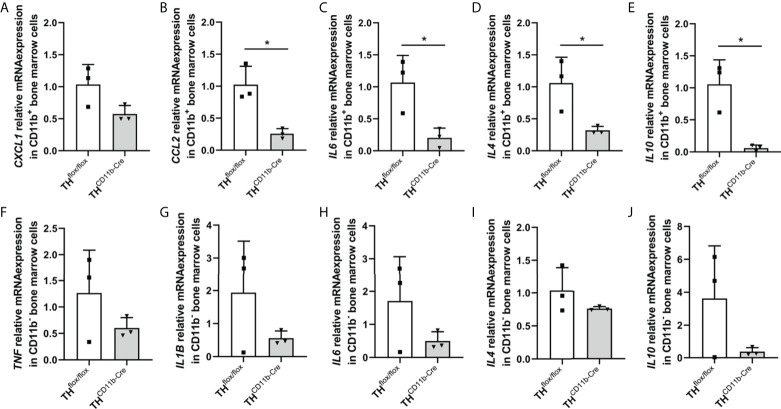
Cytokine expression in immune cells of TH^CD11b-Cre^ mice. **(A)** *CXCL1*, **(B)** *CCL2*, **(C)** *IL6*, **(D)** *IL4* and **(E)** *IL10* relative mRNA expression in CD11b^+^ bone marrow cells of twelve-week-old TH^flox/flox^ and TH^CD11b-Cre^ mice. **(F)** *TNF*, **(G)** *IL1B*, **(H)** *IL6*, **(I)** *IL4* and **(J)** *IL10* relative mRNA expression in CD11b^-^ bone marrow cells of twelve-week-old TH^flox/flox^ and TH^CD11b-Cre^ mice. n = 3; *P < 0.05.

We next analyzed the bone phenotype of twelve-week-old more mature mice. In contrast to the younger mice, we detected a significantly reduced femur and tibia length in TH^CD11b-Cre^ mice compared to TH^flox/flox^ control mice (**Figures 3A, B**). Cortical TMD was significantly reduced, while cortical thickness did not differ (**Figures 3C, D**). Trabecular TMD was also reduced, as well as trabecular bone volume to tissue volume ratio, trabecular number and thickness (**Figures 3E–H**). Trabecular separation did not significantly differ between both groups (**Figures 3I**). Histological analysis revealed a significantly reduced number and surface of osteoblasts in TH^CD11b-Cre^ mice compared to TH^flox/flox^ control mice, while osteoclast number and surface did not differ significantly (**Figures 4A–D**). Analysis of the N-terminal peptide of type I procollagen (PINP) bone formation marker in the serum did not reveal differences between TH^flox/flox^ and TH^CD11b-Cre^ mice (**Supplementary Figure 2A**). Serum concentrations of the C-terminal telopeptide of type I collagen (CTX-I) bone resorption marker were reduced in TH^CD11b-Cre^ mice compared to TH^flox/flox^ control mice (**Supplementary Figure 2B**). Immune phenotyping of these mice *via* FACS analysis demonstrated significantly higher numbers of CD11b^+^/Ly6G^+^ neutrophils in the bone marrow, while CD11b^+^/F4/80^+^ macrophage numbers were not altered (**Figures 5A, B**). Also, CD3^+^CD8^+^ as well as CD3^+^CD4^+^ T lymphocyte numbers were significantly increased in TH^CD11b-Cre^ mice compared to TH^flox/flox^ control mice (**Figures 5C, D**). RNA isolation of whole tibiae revealed a significantly reduced gene expression of CCL2, while all other measured chemokines/cytokines did not differ (**Figures 5E–H**). Further analysis of sorted bone marrow cells revealed reduced expression of CCL2, IL-6, IL-4 and IL-10 in CD11b^+^ cells (**Figures 6B–E**) and by trend reduced expression of CXCL1 **Figures 6A**), while a reduction in the expression of different cytokines/chemokines was only by trend visible in CD11b^-^ cells (**Figures 6F–J**).

**Figure 7:**
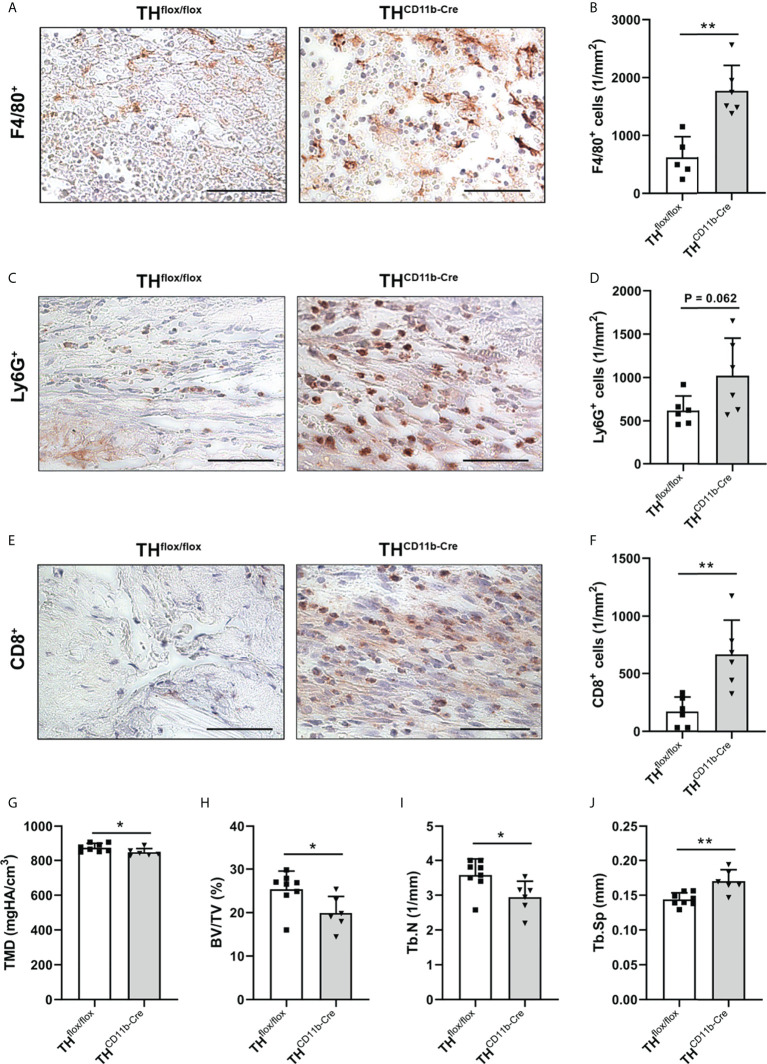
Fracture healing in TH^CD11b-Cre^ mice. Immunohistochemical staining and quantification of **(A, B)** F4/80^+^ macrophages, **(C, D)** Ly6G^+^ neutrophils and **(E, F)** CD8^+^ cytotoxic T lymphocytes in the fracture callus of TH^flox/flox^ and TH^CD11b-Cre^ mice three days post-fracture. Scale bars represent 50 µm. **(G)** Tissue mineral density (TMD), **(H)** bone volume to tissue volume ratio (BV/TV), **(I)** trabecular number (Tb.N) and **(J)** trabecular separation (Tb.Sp) in the fracture callus of TH^flox/flox^ and TH^CD11b-Cre^ mice 21 days post-fracture. n = 6-8; *P < 0.05; **P < 0.01.

To analyze the influence of the TH-KO on bone healing, TH^CD11b-Cre^ mice and TH^flox/flox^ control mice were subjected to femur osteotomy at the age of eleven weeks. Immunohistochemical staining of the fracture hematoma three days after fracture revealed significantly higher numbers of F4/80^+^ macrophages and by trend increased numbers of Ly6G^+^ neutrophils in TH^CD11b-Cre^ mice (**Figures 7A–D**). Also, CD8^+^ cytotoxic T lymphocytes were increasingly present in the fracture hematoma (**Figures 7E, F**). On day 21 after fracture, TH^CD11b-Cre^ mice displayed reduced TMD, bone volume to tissue volume ratio, trabecular number and increased trabecular separation in the fracture callus, indicating delayed fracture healing in these mice (**Figures 7G–J**).

## Discussion

Catecholamines are known to have catabolic effects on bone (8, 12, 32, 33) and to increase circulating immune cells *via* β-adrenoceptor signaling (34). In addition to the systemic synthesis in the adrenal medulla and sympathetic nerves (35), catecholamines are also produced locally in the bone marrow (BM) (36). However, it is still unknown which BM cells are exactly involved in catecholaminergic synthesis and which cells in the bone are affected by BM released catecholamines. In a previous study, we could show that CD11b^+^ myeloid cells, especially Ly6G^+^ neutrophils, at least under conditions of chronic stress, have the capacity to upregulate TH expression (26), the rate-limiting enzyme of catecholamine synthesis (28). To further investigate the role of myeloid cell-derived catecholamines on bone metabolism and regeneration as well as on the immune system, we in the current study employed a mouse line with specific knockout of TH (TH-KO) in CD11b^+^ myeloid cells (TH^CD11b-Cre^).

### Bone and immune phenotype of TH^CD11b-Cre^ mice

Bone phenotyping of eight-week-old TH^CD11b-Cre^ mice revealed only a minor effect of the TH-KO on bone development. The bone lengths of the intact femora and tibiae did not differ between TH^CD11b-Cre^ mice and TH^flox/flox^ control mice suggesting a largely unaffected long bone growth. This is supported by an unchanged distal femoral growth plate thickness and the fact that µCT analysis only revealed a significantly reduced trabecular TMD in TH^CD11b-Cre^ mice compared to TH^flox/flox^ control mice, while all other parameters were not altered. Further support for a relatively minor effect of myeloid cell-derived catecholamines on bone development and metabolism at this young age is provided by histological data, indicating neither differences in the number nor in the surface of osteoblasts and osteoclasts between the mouse lines. The same holds true for TH-KO-carrying bone marrow neutrophils and macrophages (37), for which immunohistochemistry did also not reveal any differences between the mouse lines. As osteoclasts also derive from CD11b^+^ precursor cells of the monocyte/macrophage lineage (38) and express adrenergic/dopaminergic receptors and are affected by beta-blockers and dopamine antagonists (22, 39), analysis of osteoclasts was of particular interest in the context of the current study. However, since there was no effect of the TH-KO in CD11b^+^ myeloid cells on any osteoclast parameter, direct or indirect effects on this bone cell subset seem to be unlikely. One explanation might be that we found in a previously study that TH was mainly expressed in bone marrow cells and not in osteoclasts under physiological conditions, although we did not perform double staining in that study but discriminated osteoclasts by morphology and location only (40).

Twelve-week-old TH^CD11b-Cre^ mice displayed significantly reduced femur and tibia lengths compared to TH^flox/flox^ control mice, arguing in favor of a positive effect of myeloid cell-derived catecholamines on bone metabolism and long bone growth in more mature mice. In support of this hypothesis, µCT analysis revealed a significant reduction in cortical TMD, trabecular TMD, bone volume to tissue volume ratio, trabecular number and trabecular thickness in TH^CD11b-Cre^ mice compared to TH^flox/flox^ control mice, which can be explained on a cellular level by a lower osteoblast number and surface in TH^CD11b-Cre^ mice. As the effects of the TH-KO in myeloid cells were only marginal and not significant in osteoclasts and since serum concentrations of the bone resorption marker CTX-I were even reduced in TH^CD11b-Cre^ mice compared to TH^flox/flox^ control mice, osteoblasts seem to be the main mediators of the TH-KO-induced osteopenic bone phenotype. Although we saw clearly reduced numbers and surface of osteoblasts in the femora of TH^CD11b-Cre^ mice compared to TH^flox/flox^ control mice, serum concentrations of the bone formation marker PINP did not significantly differ between both genotypes. It might therefore be interesting to additionally investigate other parts of the skeleton, like the spine, to reveal whether the observed osteopenic bone phenotype is visible in the whole skeleton. In addition to these alterations, TH^CD11b-Cre^ mice showed significantly increased numbers of Ly6G^+^ neutrophils, CD3^+^CD8^+^ cytotoxic T lymphocytes and CD3^+^CD4^+^ T helper lymphocytes in the bone marrow. Since osteoblasts derive from mesenchymal stem cells and do not belong to the myeloid lineage (41), they should not be directly affected by the TH-KO in myeloid cells. Therefore, an indirect influence of the TH-KO on bone osteoblasts and, consequently, bone phenotype is likely. One possible explanation could be that myeloid cell-derived catecholamines directly influence osteoblasts in a paracrine manner, an alternative one that the TH-KO affects the cellular metabolism of myeloid cells resulting in a compromised immune cell-osteoblast crosstalk. Support for the latter hypothesis comes from numerous studies showing that a reduction in catecholamine signaling would rather positively affects osteoblastogenesis (9, 11, 12, 14). To further analyze the effects of the TH-KO on the local bone immune system, we first isolated RNA from whole tibiae of both mouse lines. In these samples, CCL2 was significantly reduced in TH^CD11b-Cre^ mice compared to TH^flox/flox^ control mice, while all other measured cytokines did not differ significantly. Since we cannot distinguish in this analysis between the different immune cell compartments, we in a next step isolated CD11b^+^ and CD11b^-^ bone marrow cell fractions and investigated their gene expression profile. CD11b^+^ cells displayed significantly reduced gene expression of CCL2, IL-6, IL-4 and IL-10, while this was only by trend visible in CD11b^-^ cells. Based on this data and on previous studies showing that catecholamines can increase cytokine production in immune cells (42, 43), we hypothesize that myeloid cell-derived catecholamines in an autocrine manner facilitate myeloid cytokine secretion and that the reduced expression of pro-and anti-inflammatory cytokines as a consequence of the TH-KO in myeloid cells activates a feedback loop inducing proliferation of these cells. Reduced cytokine/chemokine levels might further contribute to reduced osteoblasts numbers, since CCL2 was shown to mediate mechano-responsiveness of osteoblasts and, therefore, osteogenesis (44). Further, IL-6 was demonstrated to have a dual role in osteoblastogenesis based on activation of IL-6 classic or trans-signaling, but especially during fracture healing IL-6 signaling was shown to be important for bone formation (45). Also, IL-10 is known to induce osteogenic differentiation (46), while IL-4 reduces bone formation (47). In general, immune cells are known to have a strong influence on bone homeostasis and fracture healing (27). Our results further extend this knowledge that also catecholamine expression in myeloid immune cells is important for bone health in the adult organism, although we cannot clarify the exact mechanism yet. Also, we were not able to measure catecholamine concentrations locally in the bone marrow, so we do not know which catecholamines are produced most by myeloid cells.

In conclusion, we could show an age-dependent manifestation of the TH-KO in the bone and immune phenotype of TH^CD11b-Cre^ mice. Embryonic and early childhood bone development, juvenile long bone growth and juvenile bone remodeling do not seem to be influenced by the TH-KO. In contrast, more mature mice showed a TH-KO-induced impairment of bone remodeling and long bone growth resulting in an osteopenic phenotype with reduced bone lengths. This alteration is probably due to an indirect influence of the TH-KO on the bone phenotype *via* changes in the myeloid immune phenotype. Further investigations are needed to clarify how reduced catecholamine synthesis in myeloid cells alters the proliferation, maturation and secretome of myeloid cells and how this interacts with T lymphocytes and also osteoblasts.

### Fracture healing in TH^CD11b-Cre^ mice

To further investigate the influence of the myeloid cell-specific TH-KO under inflammatory conditions, we analyzed the fracture healing process. The quantification of immune cells in the early fracture callus of TH^CD11b-Cre^ mice three days after femoral osteotomy revealed a significant increase in F4/80^+^ macrophages and CD8^+^ cytotoxic T lymphocytes, as well as a strong trend towards higher Ly6G^+^ neutrophil counts. These observations indicate an increased recruitment of immune cells of the innate and adaptive immunity to the fracture hematoma in TH^CD11b-Cre^ mice compared to TH^flox/flox^ control mice. On day 21 post-fracture, TH^CD11b-Cre^ mice displayed a significantly reduced bone mass in the fracture callus indicating an impaired fracture healing. There is a plethora of studies available showing that an imbalanced immune response after fracture is associated with disturbed bone healing (48). For example, osteoporosis and diabetes mellitus are highly prevalent diseases in western countries accompanied by systemic inflammation (49, 50) and delayed fracture healing (51, 52). Regarding macrophages, pro-inflammatory M1 and anti-inflammatory M2 subtypes are known, however it is assumed that there exist many more subclasses between these two poles (27, 53). Pro-inflammatory M1 macrophages are the dominant subtype in early phases of fracture healing switching to the anti-inflammatory M2 phenotype at later time points to resolve inflammation and to initiate bone repair (54). While M1 macrophages stimulate osteoclast bone resorption *via* secretion of pro-inflammatory cytokines like IL-1 and IL-6, M2 macrophages are known to secrete osteogenic factors like bone morphogenetic protein 2 (BMP-2) and transforming growth factor β (TGF-β), promote the differentiation of mesenchymal stem cells into osteoblasts and increase bone mineralization (55). Based on these facts, we hypothesize that the increased number of F4/80^+^ macrophages in the early fracture hematoma of TH^CD11b-Cre^ mice predominantly reflect the pro-inflammatory M1 phenotype with diminished capacity to switch to anti-inflammatory M2 macrophages leading to prolonged immune response, reduced bone formation and impaired fracture repair. However, to prove this, further analysis of the different macrophage subpopulations would be necessary. Moreover, TH^CD11b-Cre^ mice showed a strong trend of an increased number of neutrophils in the early fracture hematoma. While neutrophils are the first immune cells at the fracture site contributing to bone healing in a proregenerative way (56), excessive invasion of neutrophils is associated with impaired fracture repair (26). In addition, TH^CD11b-Cre^ mice displayed significantly increased numbers of CD8^+^ cytotoxic T lymphocytes in the early fracture hematoma compared to TH^flox/flox^ control mice, which has been described to further compromise bone healing (57). According to what has been described in patients with postmenopausal osteoporosis or rheumatoid arthritis (58), the latter might be due to the capacity of CD8^+^ T lymphocytes to secrete pro-inflammatory mediators like TNF-α, finally facilitating the activity of osteoclasts. In line with this hypothesis, depletion of CD8^+^ cytotoxic T lymphocytes improved fracture repair (57). However, as we have not investigated the detailed cytokine secretion and expression profile in the fracture callus, we cannot rule out that also in the fracture callus, immune cells secrete less cytokines/chemokines like CCL2, which might also negatively affect bone healing.

In conclusion, increased numbers of macrophages, neutrophils and cytotoxic T lymphocytes in the early fracture hematoma of male TH^CD11b-Cre^ mice are likely to at least in part mediate the impaired fracture repair. However, further studies are required to unravel in detail how myeloid cell-derived catecholamines affect the secretome of myeloid cell themselves, but also of other bone marrow leukocyte subpopulations, and how these changes exactly mediate the effects of myeloid cell-derived catecholamines on bone metabolism and regeneration described in the present study. Further, it would be interesting to investigate, by using different blockers for the distinct catecholamine receptors (adrenergic and dopaminergic) or conditional knockout mice, which catecholamines are involved in myeloid cell-bone cell crosstalk.

## Data availability statement

The raw data supporting the conclusions of this article will be made available by the authors, without undue reservation.

## Ethics statement

The animal study was reviewed and approved by Regierungspräsidium Tübingen, Germany.

## Author contributions

Design of the study: MK, MH-L, MT-M. Conduction of experiments: MK, MH-L, EK, MT-M. Analyzing data: MK, MH-L, MT-M. Interpretation of data: MK, MH-L, EK, MT-M, AI, SR. Funding of the study and supply of material: MH-L, AI, SR, HI, JV. MK, MH-L and MT-M Revising the manuscript: MK, MH-L, EK, MT-M, AI, SR, HI, JV. Approving the final manuscript: MK, MH-L, EK, MT-M, AI, SR, HI, JV. All authors contributed to the article and approved the submitted version.

## Funding

This study was supported by the German Research Foundation (CRC1149, project number 251293561, INST 40/599-1).

## Acknowledgments

We thank Tina Hieber, Iris Baum, Bettina Herde, Andrea Böhmler and Justyna Pawlak-Wurster for their excellent technical assistance.

## Conflict of interest

The authors declare that the research was conducted in the absence of any commercial or financial relationships that could be construed as a potential conflict of interest.

## Publisher’s note

All claims expressed in this article are solely those of the authors and do not necessarily represent those of their affiliated organizations, or those of the publisher, the editors and the reviewers. Any product that may be evaluated in this article, or claim that may be made by its manufacturer, is not guaranteed or endorsed by the publisher.

## Supplementary material

Supplementary Figure 1 Genotyping and verification of the *TH* knockout in TH^CD11b-Cre^ mice. Gel documentation of the **(A)** CD11b-Cre and **(B)** TH^flox^ PCR product. M = marker; bp = base pairs. TH^+^ cells among the populations of **(C)** CD11b^+^ and **(D)** CD11b^-^ cells in the bone marrow of twelve-week-old TH^flox/flox^ and TH^CD11b-Cre^ mice analyzed by FACS. ****P < 0.0001.

Supplementary Figure 2 Bone turnover markers in TH^CD11b-Cre^ mice. **(A)** PINP and **(B)** CTX-I serum concentrations in 12-week-old TH^flox/flox^ and TH^CD11b-Cre^ mice analyzed by EIA. **P < 0.01.
