# Supplementary figures and images for: Myeloid cell-derived catecholamines influence bone turnover and regeneration in mice
Source: Front Endocrinol (Lausanne). 2022 Sep 15;13:997745. doi: 10.3389/fendo.2022.997745 (PMC9520980; doi:10.3389/fendo.2022.997745)

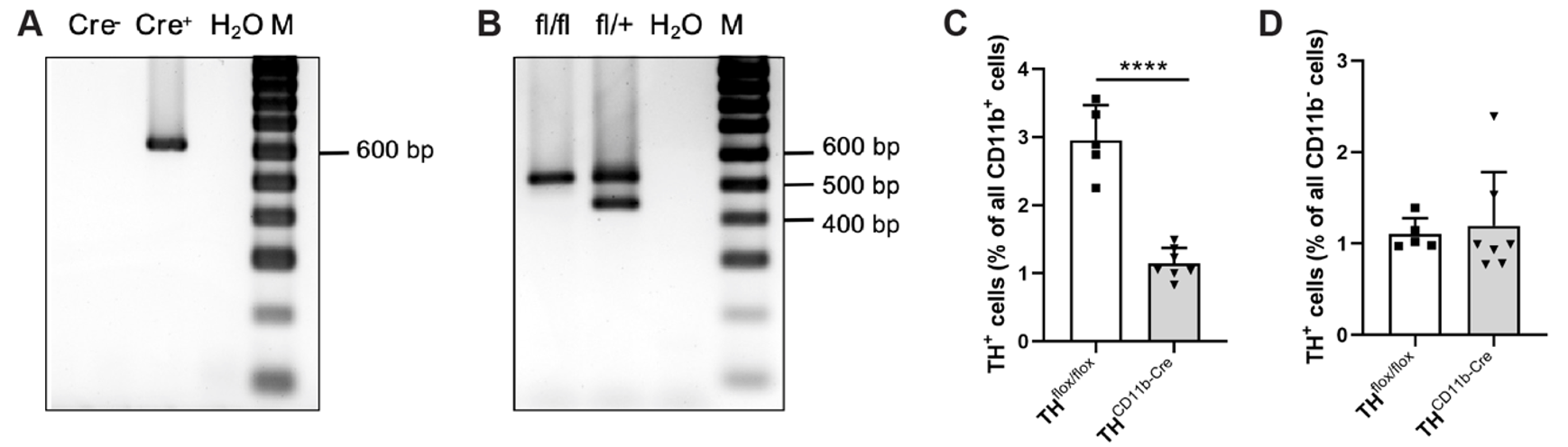

Supplement: Supplementary Figure 1 — Genotyping and verification of the TH knockout in THCD11b-Cre mice. Gel documentation of the (A) CD11b-Cre and (B) THflox PCR product. M = marker; bp = base pairs. TH+ cells among the populations of (C) CD11b+ and (D) CD11b- cells in the bone marrow of twelve-week-old THflox/flox and THCD11b-Cre mice analyzed by FACS. ****P < 0.0001. [file Image_1.jpeg]

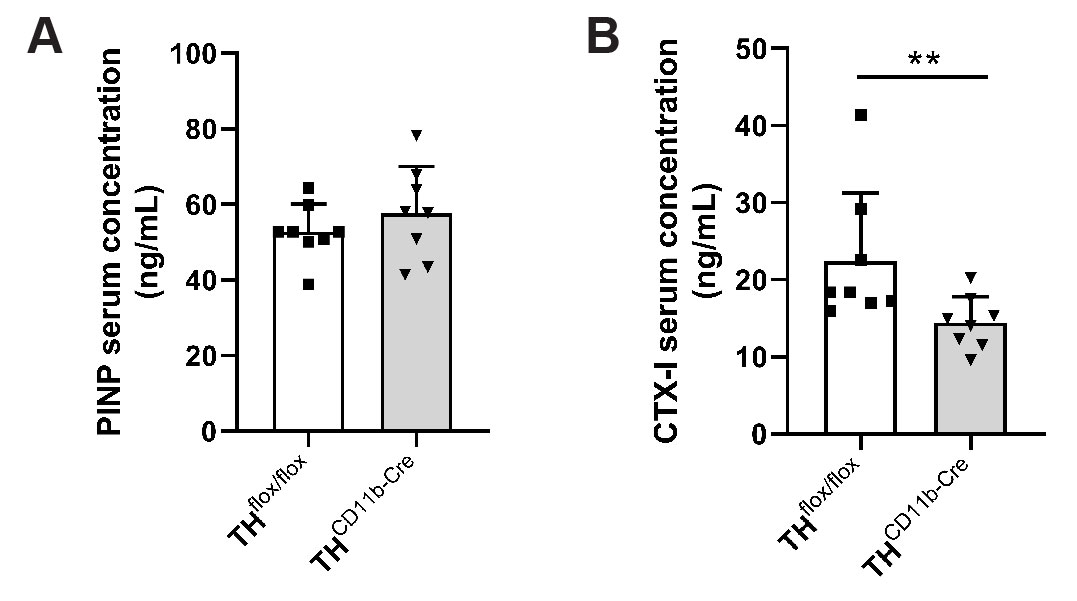

Supplement: Supplementary Figure 2 — Bone turnover markers in THCD11b-Cre mice. (A) PINP and (B) CTX-I serum concentrations in 12-week-old THflox/flox and THCD11b-Cre mice analyzed by EIA. **P < 0.01. [file Image_2.jpeg]
